# Fat droplets in the cerebrospinal fluid (CSF) spaces of the brain

**DOI:** 10.3934/Neuroscience.2024029

**Published:** 2024-11-27

**Authors:** Mark Reed, Christopher Miller, Cortney Connor, Jason S. Chang, Forshing Lui

**Affiliations:** 1 California Northstate University College of Medicine, Elk Grove, CA, USA; 2 Neurology, Kaiser Permanente South Sacramento Medical Center, Sacramento, CA, USA; 3 Clinical Sciences, California Northstate University College of Medicine, Elk Grove, CA, USA

**Keywords:** intracranial, dermoid, cyst, Hounsfield, fat, pneumocephalus

## Abstract

It is rare to find free floating fat droplets in the cerebral spinal fluid (CSF) spaces of the brain. When fat droplets are seen in the CSF spaces, the most common cause is the rupture of a dermoid cyst. Dermoid cysts are congenital inclusion cysts that form during the neural tube closure between the third and fifth weeks of embryogenesis. In this case report, we describe a case of a 74-year-old, right-handed female who presented with an acute onset of visual disturbances and left-hand numbness. Computed tomography (CT) and magnetic resonance imaging (MRI) of the head revealed hypodense “lesions” in the lateral ventricles and basal cisterns. The CT Hounsfield unit was between −41 to −83 Hounsfield Units, which is compatible with fat rather than air. The T1 weighted and FLAIR MRI showed hyperintense lesions “floating” on top of the CSF in the lateral ventricles, which is typical for fat droplets, presumably caused by a ruptured dermoid cyst. This case emphasizes the importance of analyzing Hounsfield Units to distinguish lesions by density, where fat ranges from −50 to −150 Hounsfield Units and air is −1000 Hounsfield Units. Pneumocephalus is the presence of air in the epidural, subdural, or subarachnoid space and can cause confusion, nausea, seizures and focal neurological symptoms. A careful analysis of the neuroimaging findings in the CT with or without MRI is important in making a correct diagnosis of a ruptured dermoid cyst versus pneumocephalus.

## 1. Introduction

Intracranial dermoid cysts are rare, and benign cysts are composed of both epidermal and dermal structures. These can include the abnormal proliferation of fat cells, squamous epithelium, hair follicles, and teeth, as well as sweat, apocrine, and sebaceous glands [1]. Dermoid cysts are benign congenital inclusion cysts that form from ectodermal tissue during the neural tube closure between the third and fifth week of embryogenesis [2]. As such, they typically present in patients younger than 30 years of age. Intracranial dermoid cysts are typically found in the midline, most often in the suprasellar region, though they have also been reported in locations such as the parasellar, frontonasal, and posterior fossa [2],[3]. While most are asymptomatic, some cysts may either compress the surrounding structures and cause headaches, seizures, and focal neurologic defects or develop malignant features including the transformation into a squamous cell carcinoma [3].

However, the feared complication of intracranial dermoid cysts is a rupture. The overproduction of oils from the abnormal growth of glands can cause an increasing pressure and the eventual rupture of the cyst wall. Another cause of a cyst rupture is trauma that results in direct damage to the cyst and the leakage of its contents [4]. The rupture of a dermoid cyst, either spontaneously or by trauma, will result in fat droplets in the brain CSF spaces. Fat droplets in the CSF may provoke acute meningeal inflammation, with symptoms varying from headaches and focal neurological signs to frank chemical meningitis that may present with nuchal rigidity, fever, and an altered mental status. Additionally, the rupture of cysts located in the posterior fossa can cause an obstructive hydrocephalus that manifests as headaches, an altered mental status, papilledema, and a coma [1].

## 2. Case summary

The patient is a 73-year-old, right-handed female with a past medical history of hyperlipidemia and anxiety. The patient presented early in the morning to the Emergency Department with visual disturbances and left-hand numbness. The patient's symptoms started in the evening after cleaning her trailer predominantly with her right hand with a resolution upon waking up the next morning. The visual disturbances were as if she was seeing through blue prisms. The patient denied headaches and speech or language disturbances. The patient denied weakness of the left hand, trauma to the left hand, or a prolonged pressure on the left elbow. An early stroke alert on arrival was canceled due to the patient's resolved symptoms. The vitals obtained were a blood pressure of 134/79, a heart rate of 84 beats per minute, a respiratory rate of 22, and an oxygen saturation of 98%. The physical examinations, including a neurologic exam, were normal. Her NIH Stroke Scale Score was 0. Her electroencephalogram (EEG) and routine labs were normal.

**Figure 1.**
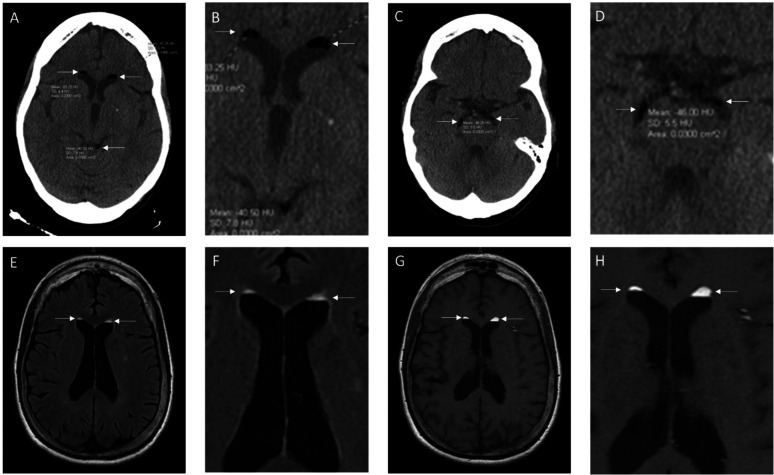
Non-contrast CT showing hypodense foci in lateral ventricles and subarachnoid space with HU (Hounsfield Units) between −40.5 and −97.25 (A,C with magnified images B,D respectively); T2 FLAIR MRI (E with magnification F) and T1 weighted MRI (G with magnification H) showing hyperintense foci in lateral ventricles overlapping foci previously seen on CT.

Computed tomography (CT) and magnetic resonance imaging (MRI) (Figure 1) of the head were performed. Her CT head revealed hypodense “lesions” in the lateral ventricles and basal cisterns, which may represent either air or fat. The CT Hounsfield unit of the lesions were between −41 to −83 Hu, which are compatible with fat and not air. The T1 weighted and FLAIR MRI revealed hyperintense lesions “floating” on top of the CSF in the lateral ventricles, which are typical for fat droplets, presumably caused by a ruptured dermoid cyst. Imaging showed no evidence of a residual cyst or hydrocephalus, and the patient was discharged with a recommendation to establish outpatient neurosurgical care. No intervention was deemed necessary.

## 3. Discussion

Dermoid cysts are rare, intracranial masses that contain a heterogeneous mixture of epidermoid and dermoid cells. Although they are benign, dermoid cysts can rupture secondary to trauma, iatrogenic complications, or the overproduction of oils within the cyst [1],[5]. A rupture of the cystic contents can cause a wide range of symptoms including focal neurologic deficits, headaches, and rarely chemical meningitis [1]. MRI imaging of a ruptured dermoid cyst often shows small, scattered foci of T1 hyperintensities with fat-fluid levels in the subarachnoid and intraventricular spaces [5]. Given the non-specific nature of the symptoms, it is imperative that a thorough examination of the neuroimaging is performed to distinguish a ruptured dermoid cyst from other pathologies that can appear similarly.

The CT scans of this patient showed well-demarcated areas of hypodense foci in the lateral ventricles and subarachnoid space, which is indicative of either air or fat. It is imperative to differentiate between the two. On initial, visual review of the CT imaging, these foci can appear to be composed of air floating above the relatively hyperdense cerebrospinal fluid. This may indicate pneumocephalus, which is defined as the presence of air in the epidural, subdural, or subarachnoid space, as well as the brain parenchyma and ventricles [6]. 75% of cases result from a neurotrauma, while the remainder of the cases can be caused by tumors, infections, and neurosurgery [6]. Pneumocephalus, while often asymptomatic, can share many clinical symptoms with a dermoid cyst rupture including headaches, confusion, dizziness, seizures, and focal neurological deficits [6]. As air and fat can appear similar on initial visual inspection of the CT neuroimaging, it is critical to further analyze the densities of the lesions to differentiate between the two.

A careful measurement of the density of our patient's lesions revealed a variable HU of −40.5 to −97.25, which is more similar to that of fat. The typical density of fat on CT imaging is roughly −50 to −100 HU; by contrast, air would have a density closer to −1000 HU [7]. The heterogeneous density of the fat droplets may be attributed to the varying lipid content of a dermoid cyst, which includes sebum (mainly fatty acids, cholesterol, and esters), keratin debris, and epidermal cells [8]–[10]. Furthermore, the foci on our patient's MRI imaging appeared hyperintense in both the T1 and T2FLAIR sequences, which is consistent with fat. Dermoid cysts can present with a variable intensity on T1 and T2-weighted images [8],[11]. Conversely, air would appear dark on an MRI.

It is also important to rule out other lesions that may appear hyperintense on both T1 and T2FLAIR imaging. Other fat-rich masses including lipomas, teratomas, and some subtypes of meningiomas and ependymomas can appear similar to dermoid cysts on imaging [2],[5]. During the late subacute phase of an intracranial hemorrhage, roughly 7–14 days post-bleed, lesions may also appear hyperintense in both the T1 and T2FLAIR sequences due to erythrocyte degradation and the presence of extracellular methemoglobin [5].

Should symptoms prove chronic or severe, determining the correct diagnosis will affect which definitive treatment should be given. For non-severe or resolving symptoms (<1 week), the treatment is similar for a ruptured dermoid cyst or pneumocephalus: observation, rest, raising the bed to 30 degrees, and analgesics [12],[13]. However, in cases of prolonged symptoms from a ruptured dermoid cyst, surgical micro-resection is necessary, especially if the components of the cyst remain attached to the dura [12],[14]. In contrast, severe symptoms from pneumocephalus would require a needle aspiration [13]. In cases where debris can obstruct the flow of the cerebral spinal fluid (CSF), a diversion with ventriculoperitoneal shunts or external ventricular devices may be used [15]. A definitive differentiation between the two conditions is necessary to accurately inform future treatment decisions and can drastically change the patient outcomes.

## 4. Conclusion

Our case illustrated the importance of a diligent review of neuroimaging when a dermoid cyst rupture is suspected. A careful examination of the CT Hounsfield Units and MRI should be performed as pneumocephalus and a dermoid cyst rupture can present very similarly on CT imaging.

## Contributions

Mark Reed: preparation and drafting of the whole manuscript and of the figure. Chris Miller: preparation and drafting of the whole manuscript and of the figure. Cortney Connor: preparation and drafting of the whole manuscript and of the figure. Jason Chang: provision, management and discussions about clinical cases and review of the manuscript. Forshing Lui: preparation and drafting of the whole manuscript and of the figure.

## Patient consent statement

Consent was obtained from the fact for publication of this case report.
